# Obtaining Fruit and Vegetables for the Lowest Prices: Pricing Survey of Different Outlets and Geographical Analysis of Competition Effects

**DOI:** 10.1371/journal.pone.0089775

**Published:** 2014-03-20

**Authors:** Amber L. Pearson, Pieta R. Winter, Ben McBreen, Georgia Stewart, Rianda Roets, Daniel Nutsford, Christopher Bowie, Niamh Donnellan, Nick Wilson

**Affiliations:** 1 Department of Public Health, University of Otago, Wellington, New Zealand; 2 Department of Geography, University of Canterbury, Christchurch, New Zealand; Newcastle University, United Kingdom

## Abstract

**Aims:**

Inadequate fruit and vegetable (F&V) consumption is an important dietary risk factor for disease internationally. High F&V prices can be a barrier to dietary intake and so to improve understanding of this topic we surveyed prices and potential competition between F&V outlet types.

**Methods:**

Over a three week early autumn period in 2013, prices were collected bi-weekly for 18 commonly purchased F&Vs from farmers' markets (FM) selling local produce (n = 3), other F&V markets (OFVM) (n = 5), supermarkets that neighbored markets (n = 8), and more distant supermarkets (n = 8), (in urban Wellington and Christchurch areas of New Zealand). Prices from an online supermarket were also collected.

**Results:**

A total of 3120 prices were collected. Most F&Vs (13/18) were significantly cheaper at OFVMs than supermarkets. Over half of the F&Vs (10/18) were significantly cheaper at nearby compared to distant supermarkets, providing evidence of a moderate ‘halo effect’ in price reductions in supermarkets that neighbored markets. Weekend (vs midweek) prices were also significantly cheaper at nearby (vs distant) supermarkets, supporting evidence for a ‘halo effect’.

Ideal weekly ‘food basket’ prices for a two adult, two child family were: OFVMs (NZ$76), online supermarket ($113), nearby supermarkets ($124), distant supermarkets ($127), and FMs ($138). This represents a savings of $49 per week (US$26) by using OFVMs relative to (non-online) supermarkets. Similarly, a shift from non-online supermarkets to the online supermarket would generate a $13 saving.

**Conclusions:**

In these locations general markets appear to be providing some substantially lower prices for fruit and vegetables than supermarkets. They also appear to be depressing prices in neighboring supermarkets. These results, when supplemented by other needed research, may help inform the case for interventions to improve access to fruit and vegetables, particularly for low-income populations.

## Introduction

Globally, inadequate consumption of fruits and vegetables (F&Vs) is a risk factor for a wide array of diseases, according to the Global Burden of Disease 2010 Study. A diet low in fruit was found to be the fifth greatest risk factor worldwide for disability and disease and a diet low in vegetables was ranked 17^th^ [1]. Still, many populations fail to meet recommended daily intakes of F&V [2], [3], [4], [5], [6]. To increase consumption, research has focused on reducing social, cultural, and environmental barriers which determine food choices [7]. Of these environmental factors, several may hold particular scope in influencing food choices including geographic access to both healthy and unhealthy food – though with inconsistent findings in the literature [5], [8], [9].

Perhaps the most consistently noted barrier to adequate consumption of F&Vs is cost [7], [10]. It is thought that cost inhibits healthy eating as energy dense, high fat foods are often cheaper than healthier fresh fruit and vegetable alternatives [7], [11]. In fact, in several wealthy countries, lower socioeconomic status households have been shown to purchase smaller volumes of F&V compared to higher socioeconomic status households [10], [12], [13]. As such, food prices may contribute to health inequalities in diet-related diseases and lower socioeconomic groups may have disproportionally high rates of these diseases. Yet evidence also suggests that reductions in price barriers influence food choices and that discounts and food subsidies increases healthy food purchasing [14]. A recent systematic review of fourteen studies on the effect of food subsidy programs on F&V consumption reported that subsidy participants had a 10–20% increased intake of targeted foods or nutrients [15]. Subsidy programs offer the potential to reduce price barriers and thus increase F&V consumption.

In addition to these programs aimed at reducing price barriers, researchers have also evaluated price differences and food choices and behaviors between types of food outlets, access to these outlets and the socioeconomic context of different outlet types. Eleven of 14 studies evaluating lower socio-economic groups estimated that food pricing strategies would be associated with pro-health outcomes [16]. In particular, studies have examined differences between low-cost and high-cost supermarkets, as well as differences between farmers' markets (FMs), where F&V are predominantly grown by the vendors, and other F&V markets (OFVMs), where the majority is not grown by the vendors. Evidence from several countries suggests that the presence of OFVM/FM in communities increases consumption of F&Vs, particularly for low-income groups [2], [3], [4], [5], [6], [17], [18]. One possible explanation is that OFVM/FMs are also thought to influence consumption by changing behaviors related to healthy food choices [3]. In addition to these benefits, the introduction of FMs into low income communities in the US has been found to lower the price of F&V sold at neighboring supermarkets by approximately 12% over a three year period [18]. However, the presence of price reductions at supermarkets neighboring OFVM/FMs has not been explored in any other settings. Furthermore, whether any such reductions temporally coincide with market days remains unexamined.

Similar to subsidy programs at supermarkets, the introduction of subsidy coupons for use at FMs has been shown to substantially increase the likelihood of purchasing F&V in those who received coupons compared to those who did not [6], [11], [17]. Importantly, it has also been shown that coupons that can only be redeemed at FMs result in a higher increase in F&V consumption than coupons redeemable at supermarkets [19]. As such, understanding differences in current pricing of F&Vs at urban supermarkets, FMs and OFVMs, as well as the geographic competition between them, can help inform the potential development of voucher programs to reduce health inequalities.

Given this background we aimed to study F&V prices in the New Zealand setting, a country that has market gardens adjacent to most of its major cities. More specifically we aimed to examine:

1. Price differences at OFVMs, FMs and supermarkets in two major cities: Christchurch and Wellington (the capital) and one national online supermarket.
2. The potential presence of a ‘halo effect’ of OFVM/FMs in stimulating F&V price reductions at nearby supermarkets relative to more distant supermarkets.
3. The potential temporal reduction of prices in supermarkets at weekends (vs mid-week), as additional potential evidence of a ‘halo effect’.
4. The estimated total price of a ‘food basket’ purchased at different outlet types so as to provide a more concrete measure of the significance of any price differences.

## Methods

### Study sites

In New Zealand, FM and OFVM are primarily located in urban centers or in peri-urban areas, as the majority of the population (>80%) live in urban areas. Supermarkets (of varying sizes) are located in most town centers or in the primary shopping area, even in relatively rural towns. Supermarkets were defined as those ‘mainly engaged in retailing groceries or non-specialized food lines’ with large annual turnover (over $1 million) [20]. Small F&V shops or specialty stores (e.g., green grocers) were excluded. This study was conducted in the urban areas of two of the country's major cities, Wellington (the capital with access to horticultural land in the region), and Christchurch (a major city surrounded by high quality farmland).

### Selection of outlets

The primary urban OFVMs and FMs were identified by an internet search, using terms including ‘farmers' market’ and ‘fruit vegetable market’. Each market (either OFVM or FM) was matched with the nearest supermarket and a distant supermarket (Table 1). Distant supermarkets were selected if they met the following criteria: the supermarket was located at least 2 km (Euclidean distance) from the OFVM/FM and was not selected as a nearby supermarket for another OFVM/FM. We selected the 2 km distance to delineate distant supermarkets as this distance approximates 20–25 minutes of walking. In choosing between all possible distant supermarkets for each OFVM/FM, we selected the distant supermarket which could be considered a realistic alternative to the OFVM/FM, based on street connectivity, terrain, and suburb division. When two distant supermarkets met these criteria, we selected the one that was the same chain as the nearby supermarket, to increase comparability (e.g., the ‘Countdown’ chain). Data were also collected from the website of the only national online supermarket in the country (as of early 2013), as an aspatial control. Thus there should not be any geographically induced competition in food prices associated with this retailer. Table 1 shows all of the selected markets and supermarkets used for food price data collection, along with the distances from the market to the corresponding supermarkets with which it was matched.

**Table 1:** Markets studied and distance from matched supermarkets in the Wellington and Christchurch regions included in this pricing study.

Market name (type)	Distance from market to the matched nearby supermarket (km)	Distance from market to the matched distant supermarket (km)
***Wellington Region***		
Hill Street (FM)	0.9	4.6
Newtown (OFVM)	0.4	3.7
Porirua (OFVM)	0.8	2.9
Riverbank Market, Lower Hutt (OFVM)	0.4	5.4
Te Papa/Waterfront (OFVM)	0.1	4.3
Victoria Street (OFVM)	1.4	4.5
***Christchurch Region***		
Opawa Market (FM)	0.7	3.9
Riccarton Bush Market (FM)	0.8	2.7

### Study period

Data were collected from the markets starting at 9.00 am for approximately 30 minutes on either a Saturday or Sunday. Data were collected from the supermarkets on the same day, before noon for 15 minutes duration. Supermarket prices were also collected on the following Wednesdays, in order to obtain a mid-week price to compare to the supermarket price on the day of the market (in the weekend). Data collection was carried out over three consecutive weeks from the 2–20 of March 2013 (early autumn in New Zealand).

### Collection of price data

F&V items were selected for inclusion based on the following criteria: (i) the item was commonly grown in New Zealand; and (ii) the item was considered relatively low-priced (based on other NZ food cost modeling [21] and national Food Price Index data).

Data were collected on the prices of apples, oranges, pears, mushrooms (field or button), onions, tomatoes, potatoes, carrots, kumara (a type of sweet potato), broccoli, cabbage, cauliflower, Chinese cabbage (‘bok choy’, ‘Shanghai bunch’), cucumber, lettuce, pumpkin, silverbeet (chard), and spinach. When prices were not given per kilogram (kg) at the outlet, these were either directly calculated from actual weights of produce or were derived by estimating weights using the standard food weight in the US Department of Agriculture online data (http://ndb.nal.usda.gov/ndb/search/list) and then calculated as a price per kg.

Market prices were collected by recording the prices from the first five F&V stalls on the left from the ‘main’ market entrance. If items were absent from stalls then further stalls were sampled, with the goal of obtaining five prices for each item. The lowest price of each item from each stall was recorded. For supermarkets, the lowest prices for each item were collected. Additional data collected on the lowest price items included whether each item was discounted, imported or organic.

### Data analyses

Price data were compiled in Microsoft Excel and then cleaned and analyzed in Stata 12.0 (College Station, TX, USA). Comparative analyses were conducted using a one-way ANOVA using the Bonferroni method for comparing mean prices for each food item between types of outlets and using a test of two proportions.

### ‘Basket’ analyses

To make the results more relevant to adults and families doing weekly shopping, we developed an idealized ‘shopping basket’ of F&V. This ‘basket’ was designed using the optimal quantities for F&V for minimizing disease risk as used in the Global Burden of Disease Study [1] (i.e., per adult: 300 g/day for fruit and 400 g/day for vegetables). As per this global study, we excluded starchy vegetables (potatoes and kumara) from the ‘basket’, as these are not favored for chronic disease risk reduction. We also excluded two items on the grounds of these being either relatively expensive at typically over $10/kg (mushrooms) or being a less commonly consumed product in the New Zealand setting (Chinese cabbage). The final ‘basket’ involved an equal division by weight of three fruits and 11 vegetables. Since detailed food wastage data are not available for New Zealand, we used values from a large United Kingdom (UK) study on food wastage (the WRAP study) [22].

### Ethics approval

University of Otago ethics approval for this study was obtained on 9 January 2013.

## Results

### Price differences by outlet

We obtained 3120 prices on the 18 F&V items (n = 24 outlets visited and one online supermarket, with bi-weekly visits for three weeks). As shown in Table 2, the mean prices of 13 of the 18 different F&V items assessed at the OFVMs were significantly cheaper than those from nearby supermarkets (mostly at the p<0.001 level). There were only two items (spinach and mushrooms) which were significantly cheaper at supermarkets than OFVMs.

**Table 2:** Fruit and vegetable prices (NZ$ per kilogram) by type of outlet (means over the three week study period in 2013).

F&V item (number of prices)	OFVM (n = 5) [O]	Farmers markets (n = 3)	Nearby super-markets (n = 8) [N]	Distant super-markets (n = 8) [D]	Online super-market (n = 1) [OS]	Differences between [N] and [D] at weekend [WE] (p-values)	Differences between [O] and all non-online supermarkets [S] (p-values)	Differences between [OS] and [S] (p-values)	Differences between [WE] and [MW] prices for [N] (p-values)	Differences between [WE] and [MW] prices for [D] (p-values)
***Fruit***										
Apples (n = 188)	1.68	3.25	2.98	3.05	2.49	0.0004 (N)*	<0.0001 (O)*	0.0874	0.2999	0.8250
Oranges (n = 169)	2.13	5.00	2.98	2.90	2.99	0.0004 (N)	<0.0001 (O)	0.1867	0.3591	0.4766
Pears (n = 180)	2.04	3.56	3.22	3.52	2.69	0.0026 (N)	<0.0001 (O)	0.1560	0.6088	0.3235
***Leafy vegetables***										
Broccoli (n = 175)	2.02	4.61	2.83	2.87	3.10	0.0001 (N)	<0.0001 (O)	0.2169	0.7012	0.1810
Cabbage (n = 172)	2.09	3.13	3.92	3.84	3.35	0.1224	<0.0001 (O)	0.2965	0.0317 (WE)*	0.3955
Cauliflower (n = 172)	2.83	3.12**	4.62	4.75	4.95	0.9417	<0.0001 (O)	0.8391	0.0207 (WE)	0.0208 (WE)*
Chinese cabbage (n = 138)	1.62	2.39**	3.42	3.64	4.75	0.0111 (N)	<0.0001 (O)	0.9593	0.8787	0.3568
Lettuce (n = 181)	4.10	8.01	7.40	7.50	6.73	<0.0001 (D)	0.1366	0.1114	0.4753	0.1114
Silverbeet (n = 163)	2.88	4.31**	5.82	6.41	4.98	0.0136 (D)	<0.0001 (O)	0.8335	0.4637	0.9094
Spinach (n = 160)	5.58	8.39**	10.79	11.07	10.15	<0.0001 (D)	0.0241 (S)	<0.0001 (S)*	0.8815	0.4714
***Starchy vegetables***										
Kumara (n = 159)	4.58	6.99	7.13	6.73	5.91	<0.0001 (D)	0.8013	0.3409	0.3112	0.6978
Potatoes (n = 187)	1.65	3.41	2.41	2.11	1.93	<0.0001 (N)	<0.0001 (O)	0.0320 (OS)	0.3512	0.8428
***Other vegetables***										
Carrots (n = 189)	1.54	4.42	2.03	2.03	1.84	<0.0001(N)	<0.0001 (O)	0.0267 (OS)	0.4655	0.4236
Cucumber (n = 188)	4.79	4.83**	7.52	7.19	6.22	<0.0001 (D)	0.7654	0.2305	0.3504	0.4689
Mushrooms (n = 143)	9.88	12.50	11.44	11.69	9.07	<0.0001 (D)	<0.0001 (S)	0.0007 (S)	0.9238	0.4409
Onions (n = 183)	1.62	4.37	2.10	2.07	1.96	<0.0001 (N)	<0.0001 (O)	0.0336 (OS)	0.5898	0.7597
Pumpkin (n = 169)	1.13	1.12**	1.46	1.58	1.26	<0.0001 (N)	<0.0001 (O)	0.0077 (OS)	0.9463	0.9999
Tomatoes (n = 204)	1.56	4.13	2.27	2.55	2.24	<0.0001 (N)	<0.0001 (O)	0.0568	0.6198	0.6807

* These codes denote the outlet or time associated with the lowest mean prices e.g., ‘N’ for nearby supermarkets (see codes in the Table headings).

** These prices were significantly cheaper than non-online supermarkets at the weekend, with p-values <0.05.

FMs prices were relatively high, but mean prices were still significantly below those of supermarkets at the weekend for six items (cauliflower, cucumber, Chinese cabbage, pumpkin, silverbeet, spinach) and there was not a significant difference in price for another six items.

There were a few significant differences in prices of items that generally favored the online supermarket over the other supermarkets (e.g., significantly cheaper potatoes, carrots, onion, pumpkin and tomatoes at the former). But at the supermarkets, spinach and mushrooms were significantly cheaper than the online supermarket. For all outlet types, the cheapest item was pumpkin. We also observed significantly more of the lowest-priced items labeled as being discounted at supermarkets compared to OFVM/FMs, despite finding significantly cheaper items at OFVMs compared to supermarkets (Table 3). We observed significantly more lowest-priced items that were organic at FMs, compared to OFVM or supermarkets, and conversely observed significantly more lowest-priced items that were labeled as being imported at supermarkets compared to OFVM/FMs and at OFVMs compared to FMs.

**Table 3:** Other characteristics of the lowest priced F&V items for which price data were collected, by outlet type.

Characteristic	OFVM (n = 5)	Farmers markets [FM] (n = 3)	Nearby super-markets (n = 8)	Distant super-markets (n = 8)	Online super-market (n = 1)	Differences between OFVM/FM and all non-online supermarkets (p-values)	Differences between OFVM and FM (p-values)
Labeled as discounted (n = 92)*	<1%	<1%	7%	3%	7%	<0.0001	0.3172
Labeled as organic produce (n = 79)	<1%	27%	0%	0%	0%	<0.0001	<0.0001
Labeled as imported (n = 111)**	2%	0%	6%	6%	6%	<0.0001	<0.0001

* That is the percentage of all F&V produce sampled for pricing data that was labeled as discounted.

** It is possible that some produce was not labeled as being from the USA, Australia or other countries from which NZ imports F&V produce.

Note: The difference in proportions for items labeled as discounted between nearby versus distant supermarkets at 7% vs 3% respectively, was significant (p<0.0001).

### Pricing ‘halo effect’

For 10 out of 18 items, there were significantly lower prices at the nearby versus distant supermarkets on the weekends (Table 2). This compared to six such differences in the opposite direction. Of note is that the prices that were significantly lower at the nearby supermarkets were for products at the lower end of the price range in which competition with the markets is likely to be most pronounced (i.e., all 10 items had mean prices of <$2.20 per kg). In contrast, all but one of the prices that were cheaper at the distant supermarkets were in the more expensive (>$4 per kg) price range.

As further evidence of a ‘halo effect’ in pricing, there were significantly lower weekend prices (relatively to mid-week prices) for two items at the nearby supermarkets vs one item at the distant supermarkets. We also found significantly more of the lowest-priced items labeled as discounted at nearby versus distant supermarkets (p<0.001).

### ‘Basket’ prices by outlet

Weekly ‘food basket’ comparisons for a two adult, two child family for ideal F&V intakes from a health perspective are shown in Table 4. These aggregate prices were (in increasing order): OFVMs (NZ$76), national online supermarket ($113), nearby supermarkets ($124), and distant supermarkets ($127), and FMs ($138). For such a family there would be savings of $49 per week by using OFVMs relative to (non-online) supermarkets. Similarly, a shift from non-online supermarkets to the online supermarket would generate a $13 saving for such a family, without considering delivery fees. Of note is the small absolute difference between the nearby and distant supermarkets (of $3), suggesting that the suspected ‘halo effect’ is having a relatively modest impact at this aggregated level.

**Table 4:** Daily and weekly costs of consuming an optimal (for health) intake of fruit and vegetables by outlet.

F&V item	Wastage factor (see *Methods*)	Daily per adult intake used in modeling (g)*	Expenditure ($ per item) using the mean price data per outlet					Estimated savings (NZ$)	
			Nearby super-markets	Distant super-markets	Online super-market	Farmers markets	OFVM	OFVM vs super-markets	Online vs other super-markets
***Fruit***		***300***							
Apples	46%	100	0.65	0.66	0.54	0.71	0.36	0.29	0.11
Oranges	40%	100	0.75	0.73	0.75	1.25	0.53	0.20	−0.01
Pears	40%	100	0.81	0.88	0.67	0.89	0.51	0.33	0.17
***Vegetables***		***400***							
Broccoli	46%	36	0.22	0.22	0.24	0.36	0.16	0.06	−0.02
Cabbage	46%	36	0.31	0.30	0.26	0.25	0.16	0.14	0.04
Carrots	46%	36	0.16	0.16	0.14	0.35	0.12	0.04	0.01
Cauliflower	46%	36	0.36	0.37	0.39	0.24	0.22	0.15	−0.02
Cucumber	46%	36	0.59	0.56	0.49	0.38	0.37	0.20	0.09
Lettuce	55%	36	0.48	0.49	0.44	0.52	0.27	0.22	0.05
Onions	46%	36	0.16	0.16	0.15	0.34	0.13	0.04	0.01
Pumpkin	46%	37	0.12	0.13	0.10	0.09	0.09	0.03	0.02
Silverbeet	55%	37	0.39	0.43	0.34	0.29	0.19	0.22	0.08
Spinach	55%	37	0.73	0.74	0.68	0.56	0.38	0.36	0.05
Tomatoes	46%	37	0.18	0.21	0.18	0.33	0.13	0.07	0.01
Adult per day			**5.90**	**6.05**	**5.38**	**6.56**	**3.63**	**2.35**	**0.60**
Adult per week			**41.30**	**42.33**	**37.64**	**45.93**	**25.40**	**16.42**	**4.18**
Two adults & two children per week**			**123.91**	**126.99**	**112.92**	**137.79**	**76.19**	**49.26**	**12.53**

Notes:

* That is 300 g of fruit and 400 g of vegetables, as per the GBD 2010 Study (see *Methods*).

** With a child assumed to have half the intake of an adult.

## Discussion

### Main results and interpretation

This study found that most of the F&Vs sampled were significantly cheaper at certain markets (OFVMs) than at supermarkets. Furthermore, it was the lower priced F&Vs that were particularly cheaper at these markets. The absolute impact of these price differences would mean that a family of four could make substantive savings ($NZ49 per week; US$26 per week) by shopping at such markets relative to supermarkets. While our ‘basket’ analyses indicated that FMs tended to provide more expensive F&Vs overall than supermarkets, one-third of items were still significantly cheaper at FMs compared to supermarkets.

In addition to price saving benefits, FMs offered significantly more consumer choice in terms of more organic products and significantly less imported products than OFVMs or supermarkets. While according to a recent systematic review there is no clear evidence for extra nutritional benefits from organic food, the review did conclude that ‘consumption of organic foods may reduce exposure to pesticide residues and antibiotic-resistant bacteria’ [23]. Another issue is whether or not the locally-produced food at FMs and OFVMs is associated with lower greenhouse gas emissions compared to supermarkets. But this issue is complex and may vary greatly depending on if produce is genuinely local or still involves large distances from the actual growing site. Finally, both of these types of markets may also have less tangible psycho-social benefits in terms of a venue for meeting other local community members and generating a link with local growers of the produce. which has been reported in New Zealand [24].

Most international research on food pricing has involved supermarkets and/or FMs. Because OFVMs are often not organized through a central body (in contrast to FMs), they may not frequently be recognized as important sources of F&Vs in the literature. In addition, OFVMs may include ‘farm stalls’ or informal places of purchasing F&Vs and may be more difficult to locate or track. However, we found these venues to be important sources of low-priced F&Vs, at least in this New Zealand context. Several studies in the USA have found cheaper F&V at farmers' markets compared to supermarkets, and one study found a mean price saving of 18% for all items [25], [26], [27].

Between supermarkets, we found there was a pattern favoring the online supermarket for lower prices, with a moderate weekly savings of $13 for a family of four. However, these saving would be eroded further with delivery costs (albeit shared with non-F&V purchases). The only foods which were significantly cheaper at the online supermarket compared to the supermarkets were potatoes, carrots, onion and pumpkin – all of which can be readily stored for longer periods of time. Interestingly, there appears to be price competition between supermarkets and other types of markets, as we found evidence for a moderate pricing ‘halo effect’ whereby the nearby supermarkets had more significantly lower F&V prices than the distant supermarkets on the weekends. On the other hand, it is possible that the nearby supermarkets could increase prices on some items if they are often not found (or only very specialty versions) at the neighboring FM. For example, both kumara and mushrooms were only observed three times at FMs. Additional evidence of a moderate ‘halo effect’ (i.e., evidence of geographical competition) came from more nearby (vs distant) supermarket items being significantly cheaper on weekends than mid-week; and nearby supermarkets offering more discounts than distant supermarkets. Nevertheless, the absolute impact of this apparent effect was not particularly large given the modest ($3 per week) difference between nearby and distant supermarkets in the ‘basket’ analysis. In comparison, one study in the USA found that the introduction of FMs lowered neighboring supermarket prices by almost 12% in three years [18].

### Study strengths and limitations

A strength of this study is the large amount of pricing data collected (n = 3120), having data collection span two different regions, and systematic collection of both weekend and mid-week data. For all markets it was feasible to select both a nearby and distant market – hence allowing an examination of possible ‘halo’ pricing effects for the first time outside of the USA to our knowledge.

Limitations to this study include the fact that quality of F&Vs was not assessed and can affect price. By selecting the lowest priced items for comparison (as part of the study aims) these data may represent prices for produce at the lower quality end of the spectrum. Additionally, the data in this study only apply to two regions of the country and for a limited time (for three weeks in early autumn). The range of F&V items studied was limited to only 18 and so does not fully represent the range of produce available (though it does cover the low-priced end of the spectrum fairly completely). We did try to include other items but these were dropped when it was apparent that they were sometimes not available in the markets (e.g., kiwifruit), and so reducing the value of outlet pricing comparisons.

A possible limitation with the pricing ‘halo’ analysis is that due to the location of the markets, the nearby supermarkets tended to be in the central city areas where residential prices are higher. This probably means that such outlets can charge higher prices relative to distant supermarkets in the suburbs, hence potentially blunting any ‘halo’ pricing effect associated with competition from markets.

It is possible that our analysis has somewhat underestimated the cost savings from shopping at markets. This is because for some items (e.g., one pumpkin or one head of broccoli) we used average weights in the analyses and yet we suspect that markets often had relatively larger such items than the supermarkets. Nevertheless, if produce from markets has higher spoilage rates relative to supermarkets, then the real cost per amount actually consumed would increase accordingly. More sophisticated future research than this unfunded study would consider such issues as spoilage rates and obtain exact weights on all items being priced.

The ‘basket’ analysis also involved various simplifying assumptions. For example, it was calculated for an ideal intake of F&Vs (by weight) whereas the actual intake for adult New Zealanders are often lower, at only 59% and 68% eating the recommended daily amount of fruit and vegetables, respectively [28]. Also, in reality, particular F&Vs are more popular (and more nutritious) than others, but these analyses treated them equally in terms of daily quantities. For example, in other work on mathematically optimizing the New Zealand diet from a low-cost, nutrition and greenhouse gas minimization perspective, we found that cabbage, carrots, Chinese cabbage, oranges and sultanas were included in the optimal mix of foods (i.e., for the diet with Mediterranean levels of F&V) [21]. Similarly, for an optimized low-cost and low-sodium diet, the items included were: carrots, Chinese cabbage, mushrooms, onions, oranges, and frozen peas (again for Mediterranean levels of F&V) [29]. Finally, wastage rates used in our ‘basket’ analysis were based on UK data and it is possible these might actually be lower for the New Zealand population. This is because New Zealanders have lower average incomes than UK citizens and so may be more attentive to minimizing waste.

### Possible implications for further research

It is ideal that there is further research around prices and access to F&Vs, especially for low-income communities. Key aspects to research include knowledge by the public that such markets are available, the time and travel costs of accessing them, the quality aspects of the F&V from markets, and the potential psycho-social benefits of visiting markets. The extent to which market produce involves lower greenhouse gas emissions (or not) is also relevant and may be highly context specific. The presence of any ‘halo’ effect on F&V prices could also be explored in other settings as more work is require to determine if observed associations involve causal pathways.

In addition to research, there is also a role for monitoring data. For example, government agencies could consider routinizing pricing data collection on F&Vs from both supermarkets and markets (e.g., the Food Price Index data on F&Vs in New Zealand is currently only from supermarkets and green grocers).

## Conclusions

This study found that most prices of commonly purchased F&Vs from OFVMs were significantly lower than those at supermarkets. Prices at FMs were however, generally higher than supermarkets, though one-third of F&Vs were still significantly cheaper. In addition, there appeared to be a moderate ‘halo’ of reduced prices at supermarkets neighboring a market, albeit somewhat less pronounced than identified in a previous US study. Online supermarkets also provided a lower price option for a select few items. These results, when supplemented by other needed research, may help inform the case for interventions to improve access to fruit and vegetables, particularly for low-income populations.
